# CD36-Mediated Lipid Accumulation and Activation of NLRP3 Inflammasome Lead to Podocyte Injury in Obesity-Related Glomerulopathy

**DOI:** 10.1155/2019/3172647

**Published:** 2019-04-11

**Authors:** Jing Zhao, Hong-liang Rui, Min Yang, Li-jun Sun, Hong-rui Dong, Hong Cheng

**Affiliations:** Division of Nephrology, Beijing Anzhen Hospital, Capital Medical University, Beijing 100029, China

## Abstract

Podocyte injury critically contributes to the pathogenesis of obesity-related glomerulopathy (ORG). Recently, lipid accumulation and inflammatory responses have been found to be involved in podocyte injury. This study is to explore their role and relationship in podocyte injury of ORG. In animal experiments, the ORG mice developed proteinuria, podocyte injury, and hypertriglyceridemia, accompanied with deregulated lipid metabolism, renal ectopic lipid deposition, activation of NOD-like receptor protein 3 (NLRP3) inflammasome, and secretion of IL-1*β* of the kidney. The expression of adipose differentiation-related protein (ADRP), CD36, sterol regulatory element-binding protein 1 (SREBP-1), and peroxisome proliferator-activated receptor *α* (PPAR*α*) in renal tissue were increased. In in vitro cell experiments, after cultured podocytes were stimulated with leptin, similar to ORG mice, we found aggravated podocyte injury, formatted lipid droplet, increased expression of ADRP and CD36, activated NLRP3 inflammasome, and released IL-1*β*. In addition, after blocking CD36 with inhibitor sulfo-N-succinimidyl oleate (SSO) or CD36 siRNA, activation of NLRP3 inflammasome and release of IL-1*β* are downregulated, and podocyte injury was alleviated. However, after blocking NLRP3 with MCC950, although podocyte injury was alleviated and release of IL-1*β* was decreased, there was no change in the expression of CD36, ADRP, and intracellular lipid droplets. Taken together, our study suggests that CD36-mediated lipid accumulation and activation of NLRP3 inflammasome may be one of the potential pathogeneses of ORG podocyte injury.

## 1. Introduction

Obesity is one of the major public health concerns with prevalence rates rapidly rising worldwide. In China, according to the 2010 China chronic disease monitoring program, the prevalence of obesity and central obesity of Chinese adults is 12.0% and 40.7%, respectively [1]. Obesity may directly lead to kidney injury, known as obesity-related glomerulopathy (ORG), and the incidence of ORG has increased concurrently with obesity [2, 3]. A retrospective study by D'Agati et al. showed that among cases that underwent renal biopsy, the percentage of ORG increased 13.5 times from 1986 to 2015 [3]. We also analyzed the cases that underwent renal biopsy in our hospital from 2010 to 2015 and calculated that the incidence of ORG with or without other kidney diseases accounted for 9.85% [4]. Podocyte injury critically contributes to the pathogenesis of ORG. Glomerular hypertrophy accompanied with podocyte hypertrophy and podocyte process fusion are the main pathological features of ORG, secondary focal segmental glomerulosclerosis may have occurred on this pathological basis, and clinical manifestations are proteinuria and progressive renal dysfunction; some patients may eventually develop end-stage renal disease (ESRD) [2, 3].

CD36, also known as a fatty acid transporter protein, is a single-chain transmembrane surface glycoprotein and belongs to the class B scavenger receptor family [5, 6]. CD36 is a multifunctional receptor that binds to two types of ligands: one is lipid-related ligands, including long-chain free fatty acids (FFA), oxidative low-density lipoprotein (ox-LDL), and oxidized phospholipids, and the other is protein-related ligands, such as advanced oxidized protein products (AOPPs), thrombin-sensitive protein-1 (TSP1), and amyloid protein [5]. In the kidney, CD36 is mainly expressed in podocytes, mesangial cells, and tubular epithelial cells. It binds to long-chain FFA ligands in podocytes and mediates lipid uptake, apoptosis, and release of reactive oxygen species (ROS) [5, 7–9].

Current studies have shown that CD36 may play an important role in kidney injury associated with metabolic diseases. The expression CD36 was upregulated in renal tissue of cases with diabetic nephropathy [7]. Sulfo-N-succimidyl oleate (SSO), an inhibitor of CD36, could alleviate albuminuria of high-fat diet-fed mice [10]. In *in vitro* experiments, after podocytes were stimulated by palmitate, a type of saturated FFA, the expression of CD36 was increased and a lipid droplet was formatted; the apoptosis of podocytes was also increased [7]. These studies suggest that CD36, which is related to lipid uptake, may play a role in podocyte injury of ORG. In addition, according to literature and our previous studies, inflammatory responses and activation of NOD-like receptor protein 3 (NLRP3) inflammasome may be involved in CD36-mediated lipid accumulation and podocyte injury [11, 12].

NLRP3 inflammasome is composed of NOD-like receptor protein 3 (NLRP3), apoptosis-related speck protein (ASC), and cysteine aspartate-1 precursor (pro-caspase-1) [13, 14]. Activation of NLRP3 results in the activation of caspase-1, which cleaves the proinflammatory cytokines IL-1*β* and IL-18 to their active forms. Mature IL-1*β* and IL-18 are released outside of the cell, resulting in a sterilized inflammatory response [14]. Numerous studies have shown that the activated NLRP3 inflammasome was involved in the pathogenesis of metabolic diseases, such as type 2 diabetes mellitus, atherosclerosis, and obesity [15, 16]. Our previous studies have observed the activation of NLRP3 inflammasome and podocyte injury in the ORG mouse model and cultured podocyte stimulated by leptin, and blocking one of the upstream receptors of NLRP3, purinergic ligand-gated ion channel 7 receptor (P2X7R), could ameliorate leptin-induced podocyte injury and inflammatory response [12].

CD36-mediated lipid accumulation may be associated with activation of the NLRP3 inflammasome. Such phenomenon was first reported in the study of arteriosclerosis [17]. OxLDL-induced IL-1*β* secretion promotes foam cell formation, which was mainly via CD36-mediated release of ROS production and activation of the NLRP3 inflammasome [18]. In terms of renal disease, Yang et al. reported that in a nephrotic syndrome animal model, increased expression of CD36 could mediate the apoptosis of podocyte through activating the NLRP3 inflammasome [11].

Is CD36-mediated lipid accumulation involved in podocyte injury of ORG? Is it associated with NLRP3 inflammasome activation? Our study showed that both in ORG mouse models and in leptin-stimulated cultured podocytes, formatted lipid droplets, increased expression of ADRP and CD36, activated NLRP3 inflammasome, and released IL-1*β* were found. In addition, blocking of CD36 reduced podocyte injury and activation of the NLRP3 inflammasome, while blocking NLRP3 could alleviate podocyte injury, but did not decrease the expression of CD36 and adipose differentiation-related protein (ADRP). Taken together, our study suggests that CD36 mediated lipid accumulation and NLRP3 inflammasome activation, which may be one of the potential pathogeneses of ORG podocyte injury.

## 2. Materials and Methods

### 2.1. Animals and Grouping

Twenty 6-week-old male C57BL/6J mice (SPF Biotechnology, China) were housed in an animal room of specific-pathogen-free cleanliness grade with 50-60% humidity at temperature 20-26°C. Mice were randomly divided into 2 groups: control group (*n* = 10), which were fed a common diet *ad libitum* that contained fat accounting for 10% kcal (Beijing Huafukang Biological Technology Co. Ltd., Beijing, China), and ORG model group (*n* = 10), which were fed a high-fat diet that contained fat accounting for 60% kcal (Research Diet, USA) as described previously [4]. All mice were sacrificed after anesthesia with pentobarbital at the end of the 12th week. One fourth of renal tissue was fixed in 4% neutral formaldehyde solution for light microscopy; one fourth of renal tissue was fixed in 2.5% glutaraldehyde solution for electron microscopy; and the renal cortex of the remaining part was rapidly preserved in liquid nitrogen for real-time quantitative polymerase chain reaction (PCR) analysis and Western blot assay.

All animal care and experimental protocols complied with the US National Institutes of Health Guide for the Care and Use of Laboratory Animals (publication no. 85-23, 1996) and were approved by the Institutional Animal Care and Use Committee of Capital Medical University (Beijing, China).

### 2.2. Biological Parameters

Body weight was measured at baseline and every 4 weeks. Kidney weight was measured after mice were sacrificed. Nocturnal 12 h urine protein was collected and measured at the 0 and 12^th^ weeks by using the Bradford protein assay kit (Beyotime Biotechnology, China) according to the user's instruction. Following sacrifice, the blood samples were collected for the measurement of serum creatinine, serum triglyceride, serum cholesterol, and blood glucose levels, as well as urine creatinine levels. The measurement was carried out by using the Olympus AU5400 Chemistry Analyzer (Olympus, Japan). The calculation methods of Lee's index, visceral fat index, and creatinine clearance rate were described as previously [19].

### 2.3. Pathological Examination

The mouse renal cortical tissues were fixed, dehydrated, embedded, sectioned (3 *μ*m), and stained with periodic acid-Schiff reagent as described [19] for light microscopy. Twenty images of glomerular maximal profiles with a vascular pole and/or urinary pole were taken under a high-power microscope (×400, Olympus, Japan) and were analyzed by Nikon NIS-Elements BR image analysis software (Nikon, Japan). The length (*μ*m) of two longest perpendicular diameters in every glomerular capillary tuft without Bowman's space was measured, and then the mean value was calculated.

The ultra-thin section renal cortical tissues were stained with uranium acetate-lead citrate for electron microscopy. Briefly, for each specimen, ten photographs (×20 000 magnification) covering different regions in the glomerular cross section were taken separately. The length (*μ*m) of the peripheral GBM was measured, and the number of slit pores overlying this GBM length was counted by Nikon NIS-Elements BR image analysis software (Nikon, Japan). The average width of the foot process was calculated as described [12].

### 2.4. Podocyte Culture and Grouping

The conditionally immortalized mouse podocyte cell line was kindly provided by Professor Maria Pia Rastaldi (S. Carlo Hospital, University of Milan). For the culture of podocytes, we followed the methods of Wang et al. [20]. Podocytes were incubated in RPMI-1640 medium (Thermo Fisher Scientific, USA) containing 10% inactivated fetal bovine serum (FBS, Thermo Fisher Scientific, USA) and 10 *μ*/mL interferon-*γ* (IFN-*γ*, Cell Signaling Technology, USA) at 33°C in humidified air with 5% CO_2_. When the cells reached 80-90% confluence, they were transferred to RPMI-1640 medium containing 10% inactivated FBS without IFN-*γ* and incubated at 37°C in humidified air with 5% CO_2_ for 10-14 days to allow differentiation. Well-differentiated podocytes were used for experiments, and different stimulants were added to the cells for different times according to experimental requirements.

### 2.5. Reverse Transcription and Real-Time Quantitative PCR

Total RNA was extracted from the mouse renal cortex tissue or cultured podocytes using TRIzol reagent (Thermo Fisher Scientific, USA) following the manufacturer's instruction. 2 *μ*g total RNA from each sample was reverse-transcribed to cDNA with EasyScript First-Strand cDNA Synthesis SuperMix (TransGen Biotech). The gene-specific primers (SBS Genetech, China) are listed in Table 1. Real-time PCR was performed using SYBR Green RT-PCR Master Mix (TransGen Biotech, China) according to the manufacturer's instruction. GAPDH was set as the internal control gene in the animal and cellular experiments. The relative quantity of mRNA expression was calculated according to the formula 2^−(targetgeneCt − GAPDHCt)^ × 10^3^, in which Ct was the threshold cycle number. All assays were repeated at least in triplicate independently.

### 2.6. Western Blot Assay

Total protein lysates were extracted from the mouse renal cortex tissue or cultured podocytes using RIPA lysis buffer (ComWin Biotech, China). Protein samples were sonicated five times for 1 s each, centrifuged at 12 000 rpm for 10 min at 4°C, and then boiled for 5 min. Protein samples were separated by 10-12% sodium dodecyl sulphate-polyacrylamide gel electrophoresis (SDS-PAGE) and transferred to nitrocellulose membranes (General Electric Co). After being blocked with 5% skim milk in phosphate-buffered saline with 0.1% Tween 20 for 1 h, the membranes were incubated with primary antibody at 4°C overnight and then incubated with secondary antibody at room temperature for 1 h. Details regarding primary and secondary antibodies are listed in Table 2. The blotted proteins were quantified using the Odyssey Infrared Imaging System (LI-COR Biosciences). *β*-Actin was set as an internal control. The relative expression level of each target protein was displayed as a ratio of target protein/*β*-actin protein. All the assays were performed at least in triplicate independently.

### 2.7. Small Interfering RNA (siRNA) of CD36

Well-differentiated podocytes were transiently transfected with 50 pmol mouse CD36 siRNA (Santa Cruz, USA) or control siRNA-A (Santa Cruz, USA) using Lipofectamine 3000 (Life technologies, USA) according to the manufacturer's instruction. The transfected cells were cultured for 24 h. The efficiency of CD36 siRNA after 24 hours of transfection was confirmed by quantitative real-time PCR of CD36 mRNA. Then, cells were incubated with fresh medium with or without leptin for another 12 h or 24 h according to real-time quantitative PCR analysis or Western blot assay, respectively.

### 2.8. Oil Red O Staining

The lipid accumulation in mouse renal tissues and cultured podocytes was evaluated by Oil Red O staining. Briefly, the sections were fixed with 4% paraformaldehyde, rinsed with 60% isopropanol, stained with Oil Red O for 20 min, and rinsed with 60% isopropanol. Finally, the samples were counterstained with haematoxylin for 5 min. The results were examined by light microscopy (Nikon, Japan).

### 2.9. Double Immunofluorescence Staining

For double staining of an indirect immunofluorescence assay of proteins and podocyte marker nephrin, frozen renal tissues of mice were fixed in 4% paraformaldehyde, cut into 5 *μ*m thick sections, permeabilized with 0.1% Triton X-100, and blocked with 2% BSA. After blocking, the sections were incubated overnight at 4°C with a rabbit monoclonal anti-ADRP (1 : 100, Abcam, UK), rabbit monoclonal anti-CD36 antibody (1 : 100, Abcam, UK), or rabbit monoclonal anti-NLRP3 (1 : 100, Novus, USA) and guinea pig polyclonal anti-nephrin (1 : 100, Progen Biotechnik, German), and then washed with PBS for three times. Next, the sections were incubated with rhodamine-labeled goat anti-mouse antibody (ZSBiO, China) and FITC-labeled rabbit anti-guinea pig antibody (Abcam, UK) for 2 h at room temperature as secondary antibodies, respectively. After staining, the tissue sections were observed with a fluorescent microscope (Nikon, Japan).

### 2.10. Statistical Analysis

All the data of continuous variables were represented as mean ± standard deviation (SD) and analyzed by using SPSS 21.0 statistical software (IBM, USA). Statistical significance between groups was analyzed by one-way ANOVA. *P* < 0.05 was considered to indicate a statistically significant difference.

## 3. Results

### 3.1. Biological Parameters and Renal Histological Changes in ORG Animal Models

The average body weight, kidney weight, Lee's index, visceral fat index, and 12 h urinary protein excretion were significantly increased in the ORG model group compared with the control group at the 12^th^ week (*P* < 0.05), while there was no significance in serum creatinine levels among the two groups (*P* > 0.05). The creatinine clearance rate (CCr) of mice in the ORG model group significantly increased than that in the control group (*P* < 0.05) (Table 3).

Hyperlipidemia was found in ORG model mice. At the end of the 12th week, levels of serum triglyceride and cholesterol in the ORG model group were significantly higher than those in the control group (*P* < 0.05). There was no significant difference in blood glucose levels between the two groups (*P* > 0.05) (Table 3).

**Figure 1:**
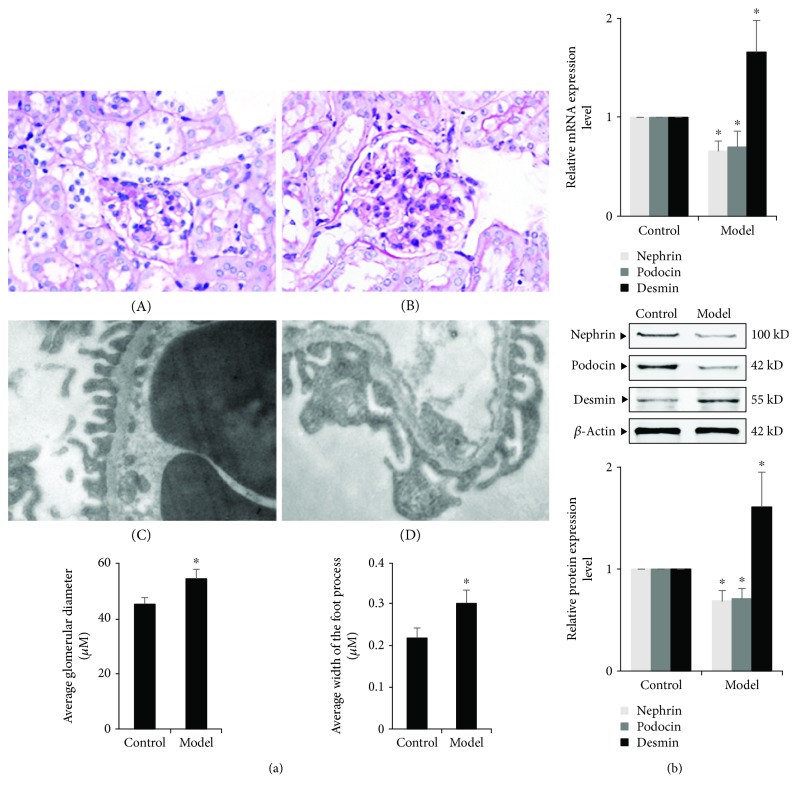
Changes in glomerular diameter and podocyte of renal tissue in the ORG model. (a) Histology of renal tissues of different groups. A and B Light microscope of PAS staining (×400). The average glomerular diameter was measured and compared. C and D, Electron microscope of glomeruli (×20000). The average width of the foot process was measured and compared. Values are represented as mean ± SD (*n* = 10). (b) The relative mRNA and protein expression levels of nephrin, podocin, and desmin of the renal cortex were measured by real-time quantitative PCR and Western blot assay. The relative protein expression level was expressed as the target protein/*β*-actin ratio. Values are represented as mean ± SD. ^∗^*P* < 0.05 vs. control group, ^#^*P* < 0.05 vs. ORG model group.

Renal tissue pathological examination showed that the mean glomerular diameter in the ORG model group was significantly longer than that in the control group at the end of the 12^th^ week (*P* < 0.05, Figure 1(a)). Under a transmission electron microscope, there was mild and segmental foot process effacement in the ORG model group, and the mean foot process width in the ORG model group was significantly wider than that in the control group at the end of the 12^th^ week (*P* < 0.05, Figure 1(a)).

### 3.2. Podocyte Injury of ORG Animal Models

We next investigated the changes of podocyte-associated molecules in renal tissues, including nephrin and podocin. The expression of desmin was also measured, which is a sensitive marker of podocyte injury [21]. Compared with the control group, the mRNA and protein expressions of podocyte-associated molecules in the ORG model group were significantly decreased, and expression of desmin was significantly increased in the renal cortical tissue of mice at the end of the 12^th^ week (*P* < 0.05) (Figure 1(b)).

### 3.3. Lipid Accumulation of Renal Tissues in ORG Animal Models

**Figure 2:**
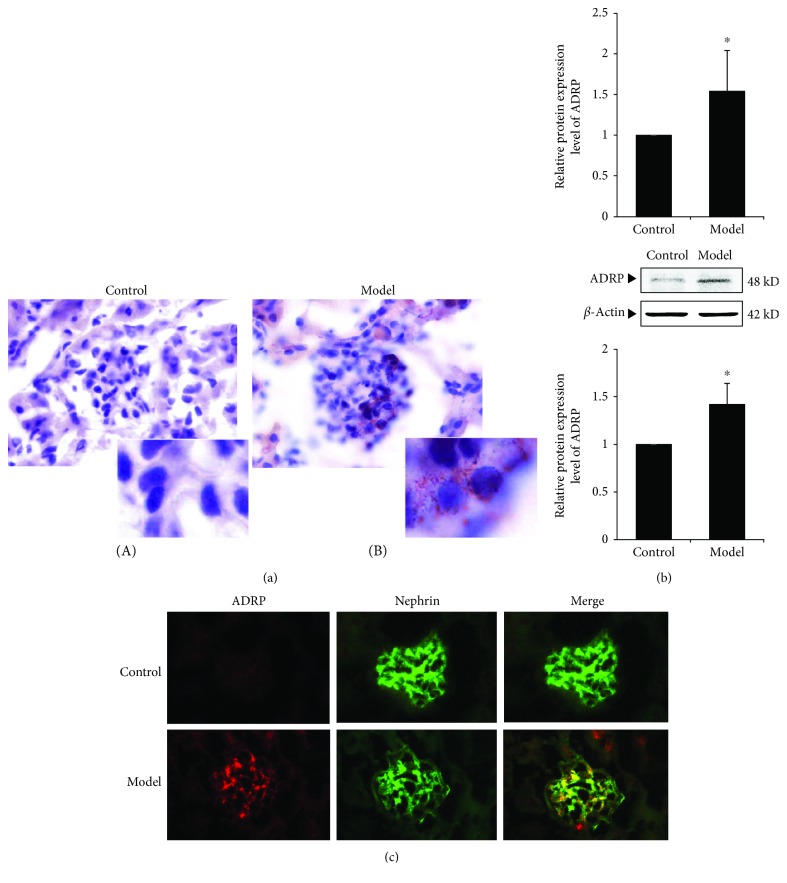
Changes of lipid accumulation of renal tissue in ORG model. (a) Representative Oil Red O staining images of renal tissue in different groups (magnification ×1000). (b) The relative mRNA and protein expression levels of ADRP of the renal cortex were measured by real-time quantitative PCR and Western blot assay. The relative protein expression level was expressed as the target protein/*β*-actin ratio. Values are represented as mean ± SD. ^∗^*P* < 0.05 vs. control group, ^#^*P* < 0.05 vs. ORG model group. (c) Double immunofluorescence staining of ADRP and nephrin of the ORG model. The localization of ADRP (red spots), nephrin (green spots), and merged image (yellow spots) in the frozen section of renal tissue of the ORG model (×400) is shown as indicated.

The results of Oil Red O staining revealed that there was an obvious lipid droplet formation in the glomeruli (Figure 2(a)). We also investigate the expression of adipose differentiation-related protein (ADRP), which is a major constituent located in the lipid droplet surface. Our results showed that the mRNA and protein expression of ADRP in the ORG model group was significantly upregulated (*P* < 0.05) (Figure 2(b)). Immunofluorescence staining of renal tissue showed that increased expression of ADRP in the glomerulus and its position overlapped with the podocyte marker protein nephrin, which suggested that there are lipid droplets in the podocytes during ORG (Figure 2(c)).

### 3.4. Expression of CD36 and Other Molecules Related to Lipid Metabolism of Renal Tissue in the ORG Model

**Figure 3:**
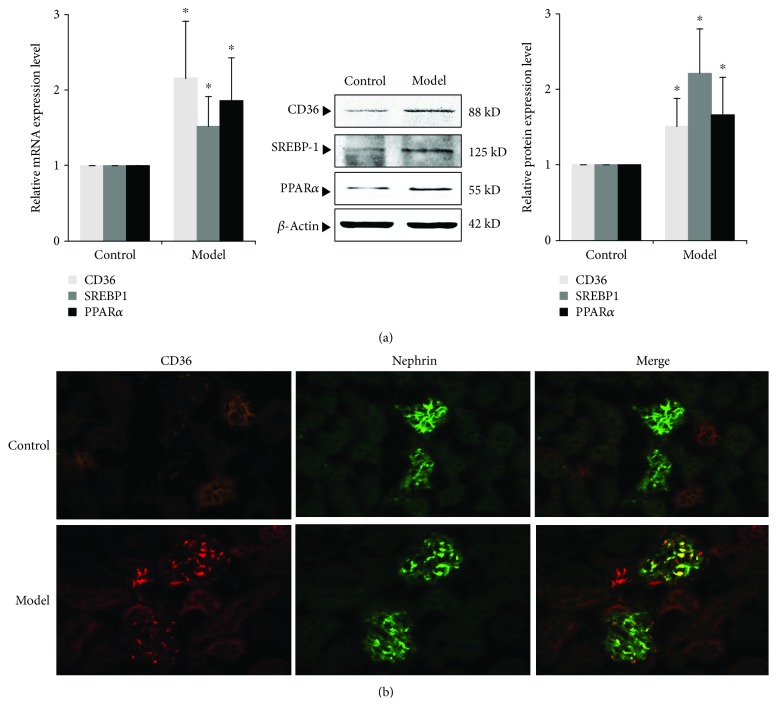
Expression of molecules related to lipid metabolism in renal tissue of ORG mice. (a) The relative mRNA and protein expression levels of CD36, SREBP1, and PPAR*α* of the renal cortex were measured by real-time quantitative PCR and Western blot assay. The relative protein expression level was expressed as the target protein/*β*-actin ratio. Values are represented as mean ± SD. ^∗^*P* < 0.05 vs. control group, ^#^*P* < 0.05 vs. ORG model group. (b) Double immunofluorescence staining of CD36 and nephrin of the ORG model. The localization of CD36 (red spots), nephrin (green spots), and merged image (yellow spots) in the frozen section of renal tissue of the ORG model (×400) is shown as indicated.

We further examined the expression changes of CD36 and other molecules associated with lipid metabolism. At the end of the 12^th^ week, the expression of CD36 was significantly increased in the ORG model group compared with the control group (*P* < 0.05). The expression of sterol regulatory element-binding protein 1 (SREBP-1), which regulates genes required for fatty acid synthesis, was significantly upregulated (*P* < 0.05). The expression of peroxisome proliferator-activated receptor *α* (PPAR*α*), the main regulator of FFA oxidation, was also upregulated (*P* < 0.05) (Figure 3(a)). The result suggests that hyperlipidemia of ORG leads to imbalance of renal lipid metabolism. Immunofluorescence staining of renal tissue showed high expression of CD36 in both renal tubules and glomeruli (Figure 3(b)). Expression of CD36 in glomerulus was overlapped with nephrin, suggesting that CD36-midiated lipid uptake and lipid accumulation increased in podocytes during ORG (Figure 3(b)).

### 3.5. Expression of NLRP3 Inflammasome and IL-1*β* of Renal Tissue in the ORG Model

**Figure 4:**
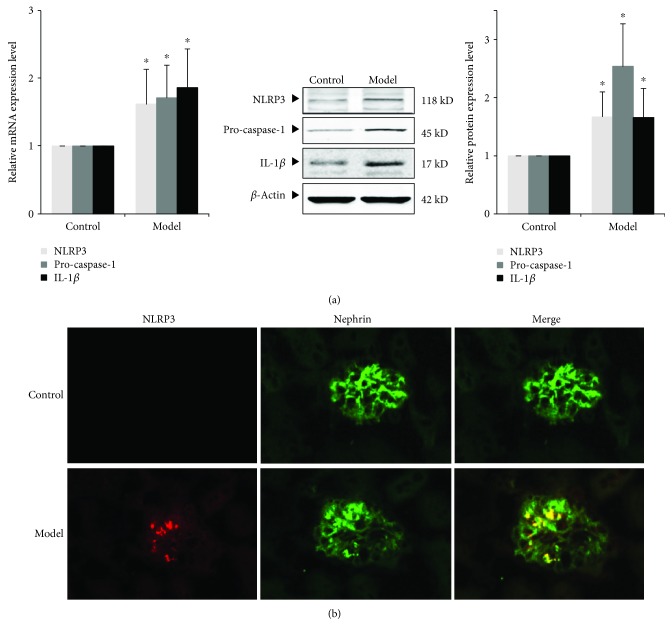
Expression of NLRP3 inflammasome and IL-1*β* in renal tissue of ORG mice. (a) The relative mRNA and protein expression levels of NLRP3, pro-caspase-1, and IL-1*β* of the renal cortex were measured by real-time quantitative PCR and Western blot assay. The relative protein expression level was expressed as the target protein/*β*-actin ratio. Values are represented as mean ± SD. ^∗^*P* < 0.05 vs. control group, ^#^*P* < 0.05 vs. ORG model group. (b) Double immunofluorescence staining of NLRP3 and nephrin of the ORG model. The localization of NLRP3 (red spots), nephrin (green spots), and merged image (yellow spots) in the frozen section of renal tissue of the ORG model (×400) is shown as indicated.

In order to detect renal inflammatory response in the ORG model, we measured the expression of components of NLRP3 inflammasome (NLRP3 and pro-caspase-1) and the downstream inflammatory factor, IL-1*β*. The results showed that the mRNA and protein expressions of NLRP3, pro-caspase-1, and IL-1*β* were significantly upregulated in the ORG model group (*P* < 0.05) (Figure 4(a)). Immunofluorescence staining of renal tissue showed a high expression of NLRP3 in the glomerulus (Figure 4(b)). Overexpressed NLRP3 overlapped with nephrin, suggesting that the overexpressed NLRP3 inflammasome exists in podocytes during ORG (Figure 4(b)).

Taken together, our results suggest that podocyte injury of ORG may be related with CD36-mediated lipid accumulation and activation of the NLRP3 inflammasome. We will next confirm the results in cellular experiments.

### 3.6. Effects of CD36 Inhibitor SSO on Leptin-Induced Podocyte Injury and Lipid Accumulation

**Figure 5:**
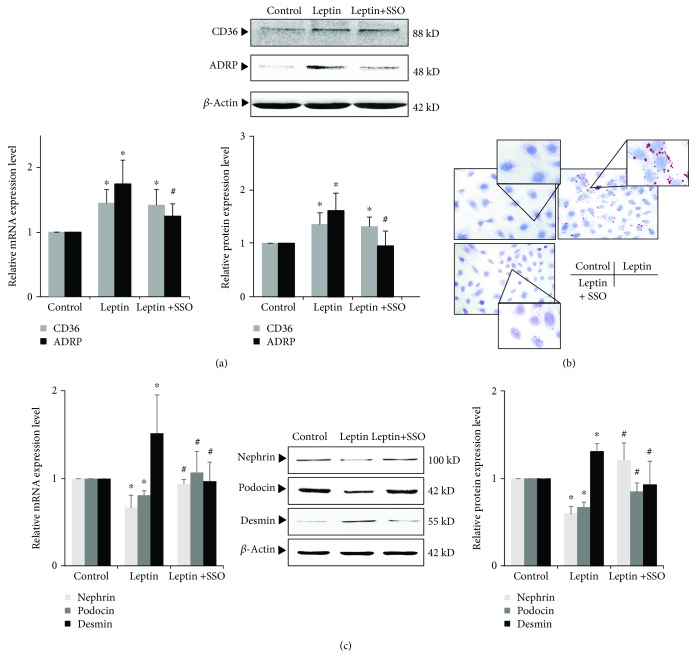
Effects of inhibiting CD36 by SSO on leptin-induced podocyte injury and lipid accumulation. (a) and (c) Differentiated podocytes were incubated in medium, medium containing 15 ng/mL leptin, or medium containing both 15 ng/mL leptin and 50 *μ*M SSO, respectively. The relative mRNA and protein expression levels of CD36 and ADRP (a) and nephrin, podocin, and desmin (c) of podocytes were measured by real-time quantitative PCR and Western blot assay. (b) Representative Oil Red O staining images of podocytes in different groups as indicated (magnification ×400). Values are represented as mean ± SD (*n* = 3). ^∗^*P* < 0.05 vs. control group, ^#^*P* < 0.05 vs. leptin + SSO group.

To mimic podocyte injury of ORG, cultured podocytes were stimulated by 15 ng/mL leptin, a key adipocytokine that regulates satiety and body fat [4, 12, 19, 22]. After leptin stimulation, the expression of CD36 and ADRP was significantly increased, and Oil Red O staining showed intracellular lipid droplet formation in podocyte (Figures 5(a) and 5(b)). The expression of nephrin and podocin is significantly downregulated (*P* < 0.05), while the expression of desmin is significantly upregulated (*P* < 0.05) (Figure 5(c)).

While when a specific inhibitor of CD36, SSO, was added, the effects of leptin on podocyte were significantly inhibited. After SSO was added, although there was no significant change in CD36 expression in the leptin+SSO group compared with the leptin group, the expression of ADRP was downregulated in podocyte (*P* < 0.05, Figure 5(a)). In addition, Oil Red O staining showed the decreased intracellular lipid droplet formation (Figure 5(b)). Compared with the leptin group, the expression of nephrin and podocin in the leptin+SSO group was significantly upregulated, and desmin expression was significantly downregulated (*P* < 0.05) (Figure 5(c)). These results suggest that inhibition of CD36 by SSO could ameliorate lipid accumulation and podocyte injury.

### 3.7. Effects of CD36 siRNA on Leptin-Induced Podocyte Injury and Lipid Accumulation

**Figure 6:**
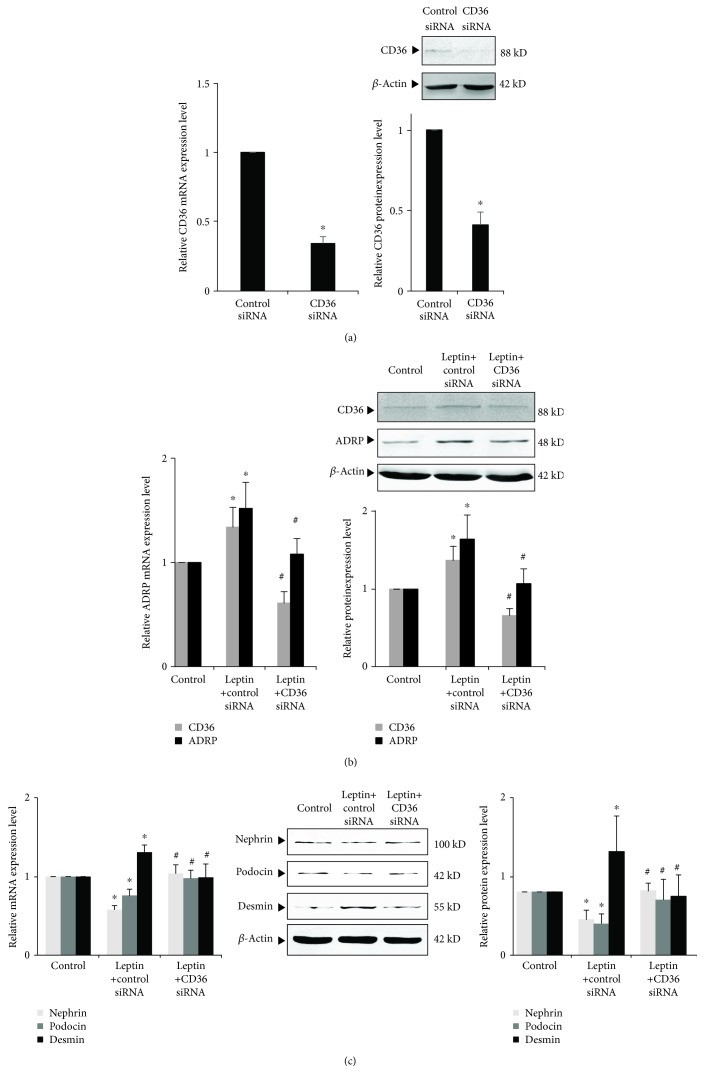
Effects of inhibiting CD36 by small interfering RNA on leptin-induced podocyte injury and lipid accumulation. (a) Differentiated podocytes were transiently transfected with 50 pmol control siRNA or CD36 siRNA, respectively. After 24 h (for mRNA) or 36 h (for protein), cells were harvested and the relative mRNA and protein expression levels of CD36 were measured by real-time quantitative PCR and Western blot assay. (b) and (c) Differentiated podocytes were transiently transfected with 50 pmol control siRNA or CD36 siRNA, respectively. After 24 h, cells were incubated in medium or medium containing 15 ng/mL leptin as indicated, respectively. Following 24 h (for mRNA) or 36 h (for protein) stimulation by leptin, the relative mRNA and protein expression levels of CD36 and ADRP (b) or nephrin, podocin, and desmin (c) of podocytes were measured by real-time quantitative PCR and Western blot assay. Values are represented as mean ± SD (*n* = 3). ^∗^*P* < 0.05 vs. control siRNA group (a) or control group (B and C), ^#^*P* < 0.05 vs. leptin + control siRNA group.

We also used CD36 siRNA to inhibit the expression of CD36 in podocyte. Our results showed that CD36 siRNA could effectively inhibit the expression of CD36 in podocyte (Figure 6(a)). Compared with the leptin + control siRNA group, the expressions of podocin and nephrin in the leptin + CD36 siRNA group were significantly upregulated and the expression of desmin was significantly downregulated (*P* < 0.05); also, the expression of CD36 and ADRP was significantly downregulated by CD36 siRNA (*P* < 0.05) (Figure 6(b)).

### 3.8. Effects of Blocking CD36 on Leptin-Induced NLRP3 Inflammasome Activation and IL-1*β* Secretion

**Figure 7:**
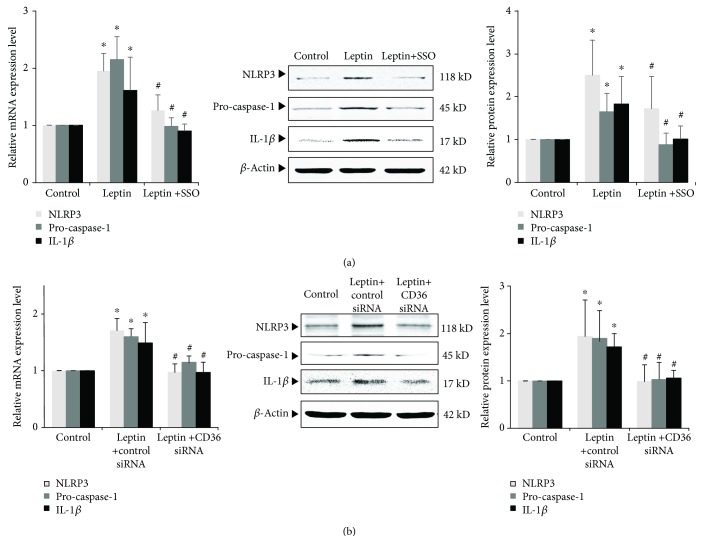
Effects of inhibiting CD36 on leptin-induced activation of NLRP3 inflammasome and IL-1*β* secretion of podocyte. (a) Differentiated podocytes were incubated in medium, medium containing 15 ng/mL leptin, or medium containing both 15 ng/mL leptin and 50 *μ*M SSO, respectively. (b) Differentiated podocytes were transiently transfected with 50 pmol control siRNA or CD36 siRNA, respectively. After 24 h, cells were incubated in medium or medium containing 15 ng/mL leptin as indicated, respectively. (a) and (b) The relative mRNA and protein expression levels of NLRP3, pro-caspase-1, and IL-1*β* of podocytes were measured by real-time quantitative PCR and Western blot assay. Values are represented as mean ± SD (*n* = 3). ^∗^*P* < 0.05 vs. control group, ^#^*P* < 0.05 vs. leptin + SSO group (a) or leptin + CD36 siRNA group (b).

We also found that stimulation of leptin could increase the expression of NLRP3 inflammasome and secretion of inflammatory cytokine IL-1*β* (Figures 7(a) and 7(b)). Could inhibition of CD36 affect such effect? Our results showed that either SSO or CD36 siRNA could significantly inhibit leptin-induced podocyte NLRP3 inflammasome activation and inflammatory factor IL-1*β* secretion. After SSO or CD36 siRNA was given, mRNA and protein expressions of NLRP3, pro-caspase-1, and IL-1*β* were significantly downregulated (*P* < 0.05) (Figures 7(a) and 7(b)). This result suggests that leptin-induced podocyte NLRP3 inflammasome activation and IL-1*β* secretion might be mediated by CD36.

### 3.9. Effects of Blocking NLRP3 Inflammasome on Leptin-Induced Podocyte Injury and Lipid Accumulation

**Figure 8:**
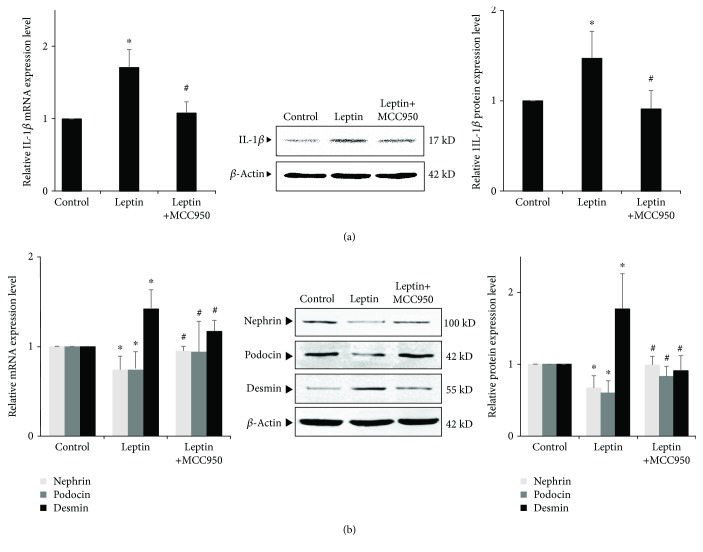
Effects of inhibiting NLRP3 by MCC950 on leptin-induced podocyte injury and IL-1*β* secretion. (a) and (b) Differentiated podocytes were incubated in medium, medium containing 15 ng/mL leptin, or medium containing both 15 ng/mL leptin and 1 *μ*M MCC950, respectively. The relative mRNA and protein expression levels of nephrin, podocin, and desmin (a) and IL-1*β* (b) of podocytes were measured by real-time quantitative PCR and Western blot assay. Values are represented as mean ± SD (*n* = 3). ^∗^*P* < 0.05 vs. control group, ^#^*P* < 0.05 vs. leptin + MCC950 group.

**Figure 9:**
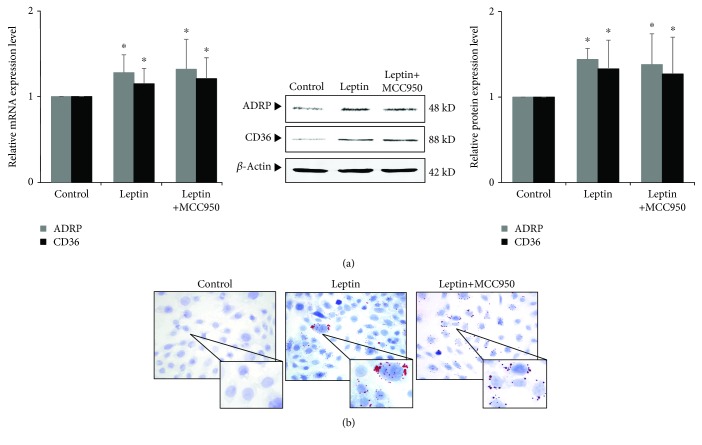
Effects of inhibiting NLRP3 by MCC950 on leptin-induced lipid uptake and accumulation of podocyte. (a) and (b) Differentiated podocytes were incubated in medium, medium containing 15 ng/mL leptin, or medium containing both 15 ng/mL leptin and 1 *μ*M MCC950, respectively. (a) The relative mRNA and protein expression levels of ADRP and CD36 of podocytes were measured by real-time quantitative PCR and Western blot assay. (b) Representative Oil Red O staining images of podocytes in different groups as indicated (magnification ×400). Values are represented as mean ± SD (*n* = 3). ^∗^*P* < 0.05 vs. control group, ^#^*P* < 0.05 vs. leptin + MCC950 group.

On the other hand, we used MCC950, a specific inhibitor of NLRP3, to observe the effects of blocking NLRP3 inflammasome on leptin-induced podocyte injury and lipid accumulation. Our results showed that after MCC950 was added, the secretion of IL-1*β* was reduced (Figure 8(a)) and leptin-induced podocyte injury was also alleviated. The expression of nephrin and podocin was significantly upregulated, and the expression of desmin was significantly downregulated (*P* < 0.05) (Figure 8(b)). However, there was no significant difference in the expression of mRNA and protein of CD36 and ADRP (*P* > 0.05) (Figure 9(a)), and Oil Red O staining also showed that lipid droplets in podocyte did not decrease (Figure 9(b)).

Our results show that although inhibition of the NLRP3 inflammasome attenuated inflammatory response and podocyte injury, it did not alter leptin-induced lipid uptake and lipid accumulation of podocytes. This result suggests that CD36-mediated lipid uptake and lipid accumulation play a vital role in podocyte injury of ORG, which might achieve such effects through CD36-mediated NLRP3 inflammasome activation and IL-1*β* secretion.

## 4. Discussion

The imbalance release of adipocytokine, including pathogenic adipocytokine (such as leptin) and protective adipocytokine (such as adiponectin), may be an important mechanism for ORG [23]. There were many studies on the role of adipocytokine in the pathogenesis of ORG [2, 23]. We also successfully established the ORG cell model by leptin-stimulated podocytes [4, 12, 19]. In recent years, the role of abnormal lipid metabolism in the pathogenesis of ORG has gradually attracted attention. This study is to observe the abnormal lipid metabolism and its pathogenesis in ORG.

ORG patients manifested metabolically unhealthy obesity, which means a central or visceral body fat distribution [24]. Verani found that obesity-associated focal segmental glomerulosclerosis or glomerulomegaly was not associated with the amount of obesity per se, but rather with serum triglycerides and renal deposition of lipid [25]. Our study also found that serum triglyceride and cholesterol levels were significantly elevated in ORG mice.

Abdominal adipose tissue is thought to generate high concentrations of circulating FFAs. Excessive FFAs are transferred and accumulated in the liver and kidney through blood circulation and formed ectopic lipid deposits [24]. de Vries et al. reported that in ORG patients, there are significant lipid accumulation and lipid droplet formation in mesangial cells and podocytes of the glomerulus [24]. We also observed lipid droplet formation and upregulated ADRP in renal tissue and podocyte of ORG mice, which suggests that there are ectopic lipid deposits and lipid accumulation in ORG.

Is lipid accumulation the causative factor of ORG? The classical “lipotoxicity” emphasizes the important role of LDL and ox-LDL, while ORG patients mainly have an elevated TG level, which means there may be different pathogenic mechanisms of ORG [24, 26, 27]. We hypothesized that CD36 may play an important role in lipid accumulation and pathogenesis of ORG.

CD36 is the main receptor for lipid uptake of podocytes. In transgenic mice overexpressing CD36 in the kidney, lipid deposition of renal tissues was significantly increased [28]. When cultured podocytes were stimulated with palmitic acid, upregulated expression of CD36, formation of intracellular lipid droplet, and release of ROS were observed [7]. Our study showed that during ORG, the expression of CD36 in renal tissue was upregulated, which was consistent with lipid droplet formation and podocyte injury. When cultured podocytes were stimulated with leptin, expressions of CD36 and ADRP were also upregulated. In addition, after blocking CD36 with inhibitor SSO or CD36 siRNA, the lipid deposition of podocytes was significantly reduced, ADRP expression was downregulated, and podocyte injury was significantly reduced. This experiment initially confirmed the important role CD36 in podocyte injury of ORG.

Renal lipid metabolism includes fatty acid intake, fatty acid synthesis, and oxidation and utilization of fatty acids [3, 29]. In an ORG mouse model, we also observed an upregulated expression of SREBP-1 and PPAR*α*. SREBP-1 is responsible for fatty acid synthesis, and PPAR*α* regulates lipid oxidation utilization [3]. It has been reported that the expression of PPAR*α* in kidney tissue is downregulated in the mouse model of high-fat diet and cases of stage IV of diabetic nephropathy [30, 31], which is inconsistent with our results. One of the possible reasons for inconsistency may be that the modeling time of ORG mice is about 12 weeks and the renal lesion seems to maintain in the early stage. So the lipid uptake and the oxidation utilization of lipid might be increased in synchronization.

We have previously observed an activation of the NLRP3 inflammasome in the ORG model and in leptin-induced podocyte injury [12]. Is there a relationship between CD36-mediated podocyte lipid accumulation and activation of NLRP3 inflammasome in ORG? Sheedy et al. reported that upregulation of CD36 promotes NLRP3 activation and IL-1*β* secretion in macrophages [17]. Liu et al. found that during the formation of foam cells in the pathogenesis of atherosclerosis, lipids cause chronic inflammatory response through CD36-mediated release of ROS and activation of the NLRP3 inflammasome [18]. Therefore, we hypothesized that CD36-mediated lipid accumulation of podocyte may lead to podocyte injury through activating the NLRP3 inflammasome and releasing IL-1*β*.

Our study showed that NLRP3 inflammasome activation and IL-1*β* secretion were increased in podocytes and renal tissue of ORG mice; after blocking CD36, lipid deposition in podocytes was reduced, activation of NLRP3 inflammasome and secretion of IL-1*β* were also inhibited, and podocyte injury was alleviated. This result suggests that CD36-mediated lipid accumulation leads to activation of the NLRP3 inflammasome and release of inflammatory factors, resulting in podocyte injury of ORG.

Previous studies have also shown that activation of the NLRP3 inflammasome may promote the expression of CD36, thereby promoting the uptake of lipids by cells. Yang et al. found that in the chronic inflammation model induced by injection of casein, the expression of CD36 in renal tissue was upregulated, and the deposition of renal fat in mice was aggravated [32]. In vitro experiments showed that mesangial cells stimulated with TNF*α* lead to upregulated expression of CD36, increased lipid uptake, and intracellular lipid accumulation [32]. Gnanaguru et al. found in macrophage studies that inhibition of NLRP3 inflammasome downregulates CD36 expression and reduces lipid uptake by cells [33]. However, our study showed that inhibition of the NLRP3 inflammasome could improve podocyte injury, but there was no significant change in CD36 and ADRP expression in podocytes, and lipid deposition was also not reduced. This result further suggests that CD36-mediated lipid uptake and lipid accumulation might be the initiating factors that promote inflammatory responses by activating NLRP3 inflammasome, leading to podocyte injury of ORG.

**Figure 10:**
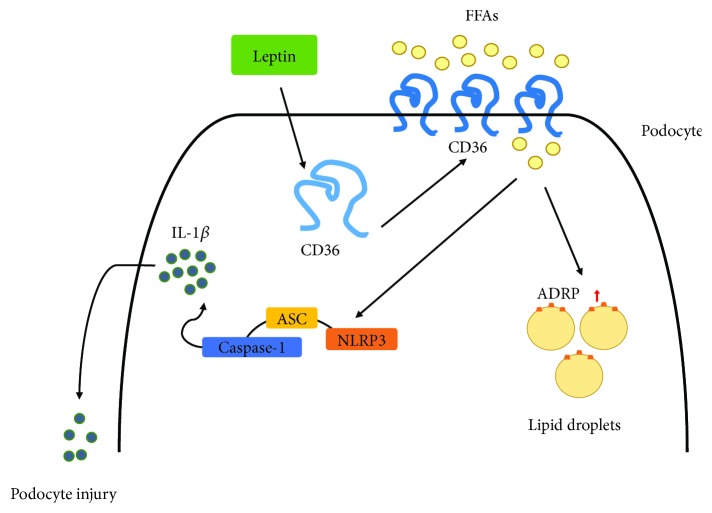
Schematic model depicting a possible mechanism that contributes to CD36-mediated podocyte injury of ORG. In the pathogenesis of ORG, after stimulation of adipocytokine such as leptin, the expression of CD36 increased, which is responsible for the FFA uptake in podocytes. Increased CD36 promotes lipid droplet formation in podocytes and further activates NLRP3 inflammasome; the cells release the mature form of inflammatory cytokines such as IL-1*β*, which causes the injury of podocytes. FFAs: free fatty acids.

Taken together, we showed that obesity and maladjusted lipid metabolism in ORG lead to renal lipid accumulation and podocyte injury, which are partially mediated by CD36; CD36-mediated lipid accumulation activates NLRP3 inflammasome, releases inflammatory factor IL-1*β*, and induces podocyte injury of ORG; inhibition of CD36 also inhibits NLRP3 activation inflammasome and ameliorates podocyte injury (Figure 10).

Different from increased CD36 and activated NLRP3 inflammasome induced by LDL and ox-LDL in atherosclerosis and foam cell formation [33–35], high-level triglycerides and FFAs conduct CD36-mediated lipid accumulation, NLRP3 inflammasome activation, and podocyte injury of ORG. In summary, we believe that CD36 may play a central role that links the pathogenesis of ORG and abnormal lipid metabolism. CD36 may become one of the important targets for ORG treatment in the future.

## Figures and Tables

**Table 1:** Primer sequences for PCR analysis in animal and cellular experiments.

Target	Primer sequence (5′-3′)	Length (bp)
Nephrin	Forward GTCTGGGGACCCCTCTATGA	209
	Reverse CAGGTCTTCTCCAAGGCTGT	

Podocin	Forward CAGAAGGGGAAAAGGCTGCT	200
	Reverse GATGCTCCCTTGTGCTCTGT	

Desmin	Forward GTTTCAGACTTGACTCAGGCAG	106
	Reverse TCTCGCAGGTGTAGGACTGG	

ADRP	Forward CTGGTGAGTGGCCTGTGT TA	199
	Reverse AAGCACACGCCTTGAGAG AA	

CD36	Forward ATGGGCTGTGATCGGAACTG	60
	Reverse AGCCAGGACTGCACCAATAAC	

SREBP-1	Forward GCGTGGTTTCCA ACATGACC	188
	Reverse TAGTGCCTCCTTTGCCACTG	

PPAR*α*	Forward CTGCAGAGCAACCATCCAGAT	70
	Reverse GCCGAAGGTCCACCATTTT	

NLRP3	Forward TCTGCACCCGGACTGTAAAC	131
	Reverse CATTGTTGCCCAGGTTCAGC	

Pro-caspase1	Forward ACAAGGCACGGGACCTATG	237
	Reverse TCCCAGTCAGTCCTGGAAATG	

IL-1*β*	Forward CGCAGCAGCACATCAACAAG	118
	Reverse GTGCTCATGTCCTCATCCTG	

GAPDH	Forward TGTGAACGGATTTGGCCGTA	202
	Reverse GATGGGCTTCCCGTTGATGA	

**Table 2:** Primary and secondary antibodies for Western blot assays.

Primary antibody	Secondary antibody
Rabbit anti-nephrin pAb (Abcam)	IRDye 800 conjugated goat anti- rabbit IgG antibody (LI-COR)
Rabbit anti-podocin pAb (Sigma)	Ditto
Rabbit anti-desmin pAb (Abcam)	Ditto
Rabbit anti-ADRP pAb (Abcam)	Ditto
Rabbit anti-CD36 pAb (Abcam)	Ditto
Rabbit anti-SREBP-1 pAb (Santa Cruz)	Ditto
Rabbit anti-PPAR*α* pAb (Santa Cruz)	Ditto
Rabbit anti-NLRP3 pAb (Novus)	Ditto
Rabbit anti-caspase1 P10 pAb (Santa Cruz)	Ditto
Rabbit anti-IL-1*β* pAb (Abcam)	Ditto
Mouse anti-*β*-actin mAb (Sigma)	IRDye 700 conjugated goat anti-mouse IgG antibody (LI-COR)

**Table 3:** Biological parameters in the different groups at the 12th week ($\bar{\text{x}}\pm \text{s}$).

Group	Control	Model
Body weight (g)	28.9 ± 1.8	31.9 ± 1.9^∗^
Kidney weight (mg)	0.34 ± 0.03	0.37 ± 0.02^∗^
Lee's index (g/cm)	16.86 ± 0.38	17.64 ± 0.56^∗^
Visceral fat index	0.02 ± 0.01	0.05 ± 0.02^∗^
Urinary protein (mg/d)	801.5 ± 178.9	1001.7 ± 230.1^∗^
Serum creatinine (*μ*mol/L)	11.3 ± 0.8	10.4 ± 1.1
Creatinine clearance rate (mL/min)	0.4 ± 0.1	0.7 ± 0.2^∗^
Serum triglyceride (mmol/L)	0.5 ± 0.1	0.9 ± 0.4^∗^
serum cholesterol (mmol/L)	2.2 ± 0.4	3.6 ± 0.3^∗^
Blood glucose (mmol/L)	9.5 ± 0.6	9.8 ± 1.3

^∗^ *P* < 0.05 vs. control group.

## Data Availability

The original data of the current study are available in the following website: https://figshare.com/s/75bd66bf6fb68b4b0106.
