# Corrigendum to “CD36-Mediated Lipid Accumulation and Activation of NLRP3 Inflammasome Lead to Podocyte Injury in Obesity-Related Glomerulopathy”

**DOI:** 10.1155/2019/8247280

**Published:** 2019-09-23

**Authors:** Jing Zhao, Hong-liang Rui, Min Yang, Li-jun Sun, Hong-rui Dong, Hong Cheng

**Affiliations:** Division of Nephrology, Beijing Anzhen Hospital, Capital Medical University, Beijing 100029, China

In the article titled “CD36-Mediated Lipid Accumulation and Activation of NLRP3 Inflammasome Lead to Podocyte Injury in Obesity-Related Glomerulopathy” [1], there were some input errors in Table 3 as the unit of kidney weight “mg,” Lee's index “g/cm,” and urinary protein “mg/d” are wrong and should be corrected as kidney weight “g,” Lee's index “g^1/3^/cm,” and urinary protein “*μ*g/d.” The corrected version of Table 3 is as follows:

The authors apologize for these errors and confirm that they do not affect the conclusions or the results of this article.

## Figures and Tables

**Table 3:** Biological parameters in the different groups at the 12th week ($\bar{x}\pm \text{s}$).

Group	Control	Model
Body weight (g)	28.9 ± 1.8	31.9 ± 1.9^∗^
Kidney weight (g)	0.34 ± 0.03	0.37 ± 0.02^∗^
Lee's index (g^1/3^/cm)	16.86 ± 0.38	17.64 ± 0.56^∗^
Visceral fat index	0.02 ± 0.01	0.05 ± 0.02^∗^
Urinary protein (*μ*g/d)	801.5 ± 178.9	1001.7 ± 230.1^∗^
Serum creatinine (*μ*mol/L)	11.3 ± 0.8	10.4 ± 1.1
Creatinine clearance rate (mL/min)	0.4 ± 0.1	0.7 ± 0.2^∗^
Serum triglyceride (mmol/L)	0.5 ± 0.1	0.9 ± 0.4^∗^
Serum cholesterol (mmol/L)	2.2 ± 0.4	3.6 ± 0.3^∗^
Blood glucose (mmol/L)	9.5 ± 0.6	9.8 ± 1.3

^∗^ *P* < 0.05 vs. control group.
